# Sex differences in the neuroinflammatory signaling pathway: effect of miRNAs on fatty acid synthesis in microglia

**DOI:** 10.1186/s13293-025-00686-8

**Published:** 2025-02-04

**Authors:** Haolin Zheng, Akiko Mizokami, Sergio Romera-Giner, Jaime Llera-Oyola, Yosuke Yamawaki, Tomomi Sano, Eijiro Jimi, Francisco García-García, Takashi Kanematsu

**Affiliations:** 1 Department of Cell Biology, Aging Science, and Pharmacology, Division of Oral Biological Sciences, Faculty of Dental Science, Kyushu University, 3-1-1 Maidashi, Higashi-Ku, Fukuoka, 812-8582 Japan; 2 OBT Research Center, Faculty of Dental Science, Kyushu University, 3-1-1 Maidashi, Higashi-Ku, Fukuoka, 812-8582 Japan; 3 Computational Biomedicine Laboratory, Prince Felipe Research Center (CIPF), 46012 Valencia, Spain; 4 Department of Advanced Pharmacology, Daiichi University of Pharmacy, 22-1 Tamagawa-Cho, Minami-Ku, Fukuoka, 815-8511 Japan; 5 Laboratory of Molecular and Cellular Biochemistry, Division of Oral Biological Sciences, Kyushu University, 3-1-1 Maidashi, Higashi-Ku, Fukuoka, 812-8582 Japan

**Keywords:** Fatty acid synthesis, Inflammation, Microglia, miRNA, Sex differences, Testosterone

## Abstract

**Background:**

Significant sex differences exist in the prevalence and incidence of Alzheimer’s disease (AD). Notably, testosterone has been reported to regulate cognitive functions in the brain, with low serum testosterone levels correlating with increased AD risk. However, the specific mechanisms underlying this relationship remain unclear. Recent studies have demonstrated that microglia, the primary innate immune cells in the brain, play a crucial role in AD development. Therefore, this study aimed to explore sex differences in microglial function, specifically focusing on the role of testosterone in miRNA-mediated regulation of microglial gene expression.

**Methods:**

Microglia were isolated from pooled hippocampal tissue of five 8-month-old male and female mice. Total RNA was extracted and subjected to miRNA microarray analysis. The mouse microglial cell line MG6 was used for in vitro experiments. Following testosterone treatment, miRNA, gene, and protein expression levels were investigated. An inflammatory response was induced using lipopolysaccharide (LPS) stimulation, and subsequent p65 phosphorylation was assessed.

**Results:**

Sex-dependent differences were observed in miRNA-mediated biological processes, with males exhibiting greater changes. Male-enriched miRNAs were associated with fatty acid synthesis and metabolism pathways. In MG6 cells, testosterone treatment upregulated the expression of several miRNAs enriched in male microglia, particularly those targeting genes related to fatty acid synthesis. Additionally, testosterone significantly reduced the gene expression of fatty acid synthase (FASN). This testosterone-induced inhibition of FASN expression attenuated NF-κB/p65 phosphorylation. Consequently, the suppression of FASN expression led to reduced expression and secretion of tumor necrosis factor-alpha following LPS stimulation in MG6 cells.

**Conclusions:**

These findings suggest that testosterone modulates inflammation in male microglia by regulating fatty acid synthesis, potentially contributing to the observed sex differences in AD pathogenesis.

**Supplementary Information:**

The online version contains supplementary material available at 10.1186/s13293-025-00686-8.

## Highlights

- miRNA expression in hippocampal microglia exhibits sex-dependent differences, with males exhibiting greater changes.
- Testosterone upregulated the expression of several male-enriched miRNAs and downregulated fatty acid synthase (FASN) levels.
- Testosterone-induced inhibition of FASN levels reduced p65 phosphorylation in the NF-κB inflammatory pathway and decreased TNF-α secretion after lipopolysaccharide stimulation.
- Testosterone modulates inflammation in microglia by regulating fatty acid synthesis, potentially contributing to the sex differences in AD pathogenesis.

**Supplementary Information:**

The online version contains supplementary material available at 10.1186/s13293-025-00686-8.

## Plain Language summary

Sex differences in Alzheimer’s disease (AD) are well-established, with females experiencing a higher prevalence and greater disease severity. Microglia, the primary innate immune cells of the brain, play a key role in AD development. Notably, sex-specific differences in microglial gene expression and function, including the regulatory roles of microRNAs (miRNAs), have been documented and may contribute to the differential pathogenesis of AD between sexes. This study investigated sex-specific differences in miRNA expression patterns. miRNA microarray analyses of hippocampal microglia from adult male and female mice revealed distinct miRNA expression patterns, with the potential target genes of male-enriched miRNAs being associated with pathways involved in fatty acid biosynthesis. Testosterone stimulation of the mouse microglial cell line MG6 resulted in the upregulation of several male-enriched miRNAs, leading to downregulated fatty acid synthase (FASN) expression. This testosterone-mediated suppression of FASN levels subsequently inhibited NF-κB/p65 phosphorylation. These findings highlight the regulatory role of testosterone in the NF-κB inflammatory signaling pathway through de novo fatty acid synthesis. Our study suggests that testosterone, through the upregulation of specific miRNA expression, plays a crucial role in suppressing inflammatory responses in male microglia, contributing to sex-specific characteristics under inflammatory conditions and potentially explaining the lower susceptibility to AD observed in males.

**Supplementary Information:**

The online version contains supplementary material available at 10.1186/s13293-025-00686-8.

## Background

Accumulating evidence indicates that sex differences exist in numerous diseases, as well as variations in disease onset, progression, and prognosis [1]. However, therapeutic and preventive strategies have often been developed without considering these disparities. Such an approach may not only lead to ineffective outcomes but could also be detrimental to specific patient populations.

Alzheimer’s disease (AD), the most prevalent form of dementia, is characterized by the abnormal extracellular accumulation of amyloid-β and the formation of intracellular neurofibrillary tangles composed of hyperphosphorylated tau protein in the brain. These pathological changes trigger neuroinflammation, neurodegeneration, and cognitive decline [2–4]. Despite adjusting for longer life expectancy, the prevalence of AD is found to be significantly higher in females, constituting approximately two-thirds of all patients with AD [5]. Epidemiological studies have demonstrated that females experience a greater incidence, severity, and progression of AD, with this disparity becoming increasingly pronounced with advancing age [6–9]. Sex differences in AD are not limited to humans; several AD mouse models, including 3xTg-AD and 5xFAD mice, also exhibit disparities in disease outcomes based on sex [10–12]. In 5xFAD mice, females exhibited higher levels of amyloid-β accumulation and heightened inflammation, accompanied by deficits in object and social exploration compared to their male counterparts [12]. Similarly, female 3xTG-AD mice displayed higher levels of amyloid-β in the brain and a marked decline in cognitive function compared to males [10].

Microglia are resident immune cells in the central nervous system, which play a pivotal role in the progression of AD owing to their ability to release cytokines and clear aggregated proteins such as amyloid-β. The aggregation and accumulation of amyloid-β trigger hyperactivation of microglia, resulting in the upregulation of the expression of proinflammatory cytokines, including tumor necrosis factor-alpha (TNF-α), interleukin-1β, and interleukin-6, all of which can ultimately induce neuronal damage [13]. Therefore, sex differences in the characteristics of microglia may contribute to the observed sex differences in AD.

A series of studies have identified notable sex differences in the properties of microglia. In rats, the number and morphology of microglia throughout development exhibit clear variations between sexes [14]. Similarly, Villa et al*.* demonstrated significant sex differences in the transcriptomes of microglia isolated from healthy adult male and female mice [15]. Male microglia exhibit a more proinflammatory phenotype, likely established by the androgen surge during the neonatal period [15]. Furthermore, the expression profiles of microglial microRNAs (miRNAs) have been demonstrated to be influenced by sex, both in the basal state and in tauopathy [16]. miRNAs are small noncoding RNAs that regulate gene expression post-transcriptionally and are implicated in various cellular processes, including inflammation, lipid metabolism, and neuronal function [17]. Notably, studies involving the depletion of the miRNA-processing enzyme Dicer have demonstrated that miRNA-mediated regulation of immune-related gene expression significantly affects male microglia more than female microglia [16].

This study hypothesized that miRNA-mediated regulation of microglial gene expression in males may play a role in suppressing microglia-mediated neuroinflammation, potentially providing a protective effect against AD onset. Therefore, this study aimed to investigate how sex differences in miRNAs influence the inflammatory response of microglia. Several male-enriched miRNAs were identified as potential regulators of genes associated with fatty acid synthesis, including the fatty acid synthase (FASN) gene*.* Notably, the expression of certain miRNAs was upregulated in response to testosterone stimulation, leading to a subsequent downregulation of *Fasn* expression. Our findings demonstrated that testosterone stimulation attenuated the nuclear factor-κB (NF-κB) inflammatory signaling pathway by suppressing FASN-dependent de novo lipogenesis in microglia, potentially contributing to the observed sex differences in AD.

## Materials and methods

### Animals

Ten 8-month-old male and female C57BL/6 J mice were housed in groups of five per cage in a specific pathogen-free facility, maintained under a 12 h light/12 h dark cycle, and provided ad libitum access to food and water. The oestrus cycle was not monitored in female mice. All animal experiments were approved by the animal ethics committee of Kyushu University (approval nos. A19-316-1 and A21-136-0).

### Isolation of microglia

Hippocampal microglia were isolated using a magnetic-activated cell sorting system (Miltenyi Biotec, Teterow, Germany), as described previously [18]. Briefly, the hippocampi isolated from male and female mice (five mice per group) were pooled and minced in Hank’s balanced salt solution (HBSS, Fujifilm Wako, Osaka, Japan). A neural tissue dissociation kit (Miltenyi Biotec) was employed for enzymatic digestion to obtain single cells. Following the removal of tissue debris using a 70-µm cell strainer, myelin was eliminated using Myelin Removal Beads II (Miltenyi Biotec) and a magnetic LS column (Miltenyi Biotec). Subsequently, microglia were isolated from the eluted cells labeled with CD11b MicroBeads using a magnetic MS column (Miltenyi Biotec).

### miRNA microarray analysis

Total RNA was isolated from freshly isolated hippocampal microglia using TRIzol Reagent (Invitrogen, Waltham, MA, USA) and subsequently purified using the miRNeasy Mini Kit (QIAGEN, Hilden, Germany) according to the manufacturer's instructions. The RNA samples were quantified using an ND-1000 spectrophotometer (NanoDrop Technologies, Wilmington, DE, USA), and their quality was confirmed using an Experion System (Bio-Rad Laboratories, Hercules, CA, USA) or Agilent 4200 TapeStation (Agilent Technologies, Santa Clara, CA, USA). Subsequently, 100 ng of total RNA from each sample was labeled using a FlashTag Biotin HSR RNA Labeling Kit (Thermo Fisher Scientific, Waltham, MA, USA) and hybridized to an Affymetrix GeneChip miRNA 4.0 Array (Applied Biosystems, Waltham, MA, USA) according to the manufacturer's instructions. All hybridized microarrays were scanned using an Affymetrix scanner (Affymetrix, Santa Clara, CA, USA). Relative hybridization intensities and background hybridization values were calculated using Affymetrix Expression Console (Affymetrix).

### Bioinformatic analysis of miRNA microarray data

miRNAs exhibiting a relevant log fold change threshold (absolute values >  ± 2) identified through differential expression analysis were selected for further functional enrichment. The DIANA mirPath webtool [19] was used to identify the Kyoto Encyclopedia for Genes and Genomes (KEGG) pathways and Gene Ontology (GO) term entries that were significantly targeted by the selected miRNAs. The mirPath server was used to conduct enrichment analysis through a posteriori analysis, calculating the significance levels (p-values) for each miRNA in relation to every functional feature. Subsequently, using Fisher’s meta-analysis method, a merged p-value was extracted for each functional feature through the combination of the previously calculated significance levels. The final p-value represents the probability of a particular functional feature being significantly enriched by the genes targeted by the selected miRNAs. To elucidate the relationships among the functional features of the genes, an enrichment map plot was generated using the clusterProfiler R package (v4.0) [20]. The enrichment map plot visually represents the enriched terms as a network, with the functional features in which they participate depicted as nodes with different sizes and colors based on the number of overlapping genes and their corresponding levels of significance.

### Reverse transcription and quantitative miRNA expression analysis

RNA enriched in miRNAs was extracted using a miRNeasy Mini Kit (QIAGEN). A MicroScript microRNA cDNA Synthesis Kit (Norgen Biotek Corp., Ontario, Canada) was used for reverse transcription. The abundance of miRNA was quantified through quantitative PCR (qPCR) using THUNDERBIRD Next SYBR qPCR Mix (Toyobo, Osaka, Japan), in conjunction with the universal PCR reverse primer (Norgen Biotek Corp.) and a specific forward primer designed from the whole sequence of the miRNAs of interest. RNU6B expression served as an internal control.

### Quantitative real-time PCR

Total RNA was subjected to reverse transcription using a High-Capacity cDNA Reverse Transcription Kit (Life Technologies, Carlsbad, CA, USA). qPCR was performed using THUNDERBIRD Next SYBR qPCR Mix (Toyobo) on a StepOnePlus Real-Time PCR system (Thermo Scientific). 18S rRNA expression served as an internal control. Primer sequences were obtained from PrimerBank (https://pga.mgh.harvard.edu/primerbank/index.html) and are provided in Table S1.

### Cell culture and reagents

Mouse microglial MG6 cells (Riken Cell Bank, Ibaraki, Japan) were cultured in high-glucose Dulbecco’s modified Eagle’s medium (Nacalai Tesque Inc., Kyoto, Japan) supplemented with 10% fetal bovine serum (FBS), 10 µg/mL insulin (Cell Science & Technology Institute Inc., Sendai, Japan), 0.1 mM 2-mercaptoethanol (Sigma‒Aldrich, St. Louis, MO, USA), penicillin (100 U/mL), and streptomycin (0.1 mg/mL) under 5% CO_2_ at 37 °C. The cells were monitored for growth and morphological characteristics using an inverted microscope and passaged upon attaining a minimum confluency of 80%. The cells were cultured in the medium containing charcoal-stripped FBS (Serana, Brandenburg, Germany) for 16 h before testosterone stimulation. Testosterone (Sigma-Aldrich) was dissolved in 100% ethanol and added to the culture medium containing charcoal-stripped FBS at a final ethanol concentration of 0.1%. Lipopolysaccharide (LPS) from *Escherichia coli* (Sigma-Aldrich) was dissolved in phosphate-buffered saline and applied at a concentration of 1 ng/mL. C75 trans (Selleck Chemicals, S8915, Houston, TX, USA) was dissolved in dimethyl sulfoxide and added to the culture medium at the indicated concentrations 24 h before stimulation. TNF-α secreted into the culture medium was quantified using a mouse TNF-α ELISA MAX Standard kit (BioLegend, San Diego, CA, USA) according to the manufacturer’s instructions.

### Transfection of miRNA mimics and inhibitors

For the transfection of miRNA mimics, MG6 cells were seeded into 6-well plates at a density of 5 × 10^5^ cells/well. After overnight culture, control or miRNA mimics (Ajinomoto Bio-Pharma Services, Osaka, Japan) were transfected at the final concentration of 1 nM using INTERFERin siRNA Transfection Reagent (Polyplus, Illkirch, France) according to the manufacturer’s instructions. For the transfection of miRNA inhibitors, MG6 cells were seeded into 6-well plates at 1 × 10^5^ cells/well. After overnight culture, control or mix of miR-125a-5p and miR-339-5p inhibitors (Bioneer Corporation, Daejeon, Korea) was transfected at the final concentration of 10 nM each using INTERFERin siRNA Transfection Reagent according to the manufacturer’s instructions.

### Western blot

Western blot analysis was performed as described previously [21]. Briefly, cell lysates were prepared using radioimmunoprecipitation assay buffer (Fujifilm Wako) supplemented with protease and phosphatase inhibitor cocktails (Nacalai Tesque Inc.). Protein concentrations were determined with a Protein Assay BCA Kit (Fujifilm Wako). Next, equal amounts of protein were separated using polyacrylamide gel electrophoresis and subsequently transferred onto a polyvinylidene difluoride membrane (Merck-Millipore, Billerica, MA). After blocking with Blocking-One or Blocking-One P solution (Nacalai Tesque Inc.), the membranes were incubated with primary antibodies overnight at 4 °C, followed by incubation with horseradish peroxidase-conjugated secondary antibodies for 1 h at 20–25 °C. The antibodies used are listed in Table S2. Blots were visualized using ImmunoStar LD (Fujifilm Wako), and digital images were captured using ImageQuant LAS 4000 mini (GE Healthcare, Chicago, IL, USA). Band intensities were quantified using ImageJ software (National Institutes of Health, Bethesda, MD, USA).

### Statistical analysis

Quantitative data are presented as the mean ± standard deviation (SD). Statistical analyses were performed using JMP Pro 16.0.0 (SAS Institute Inc., Cary, NC, USA).

## Results

### Male-enriched miRNAs were associated with fatty acid synthesis and metabolism pathways

**Fig. 1:**
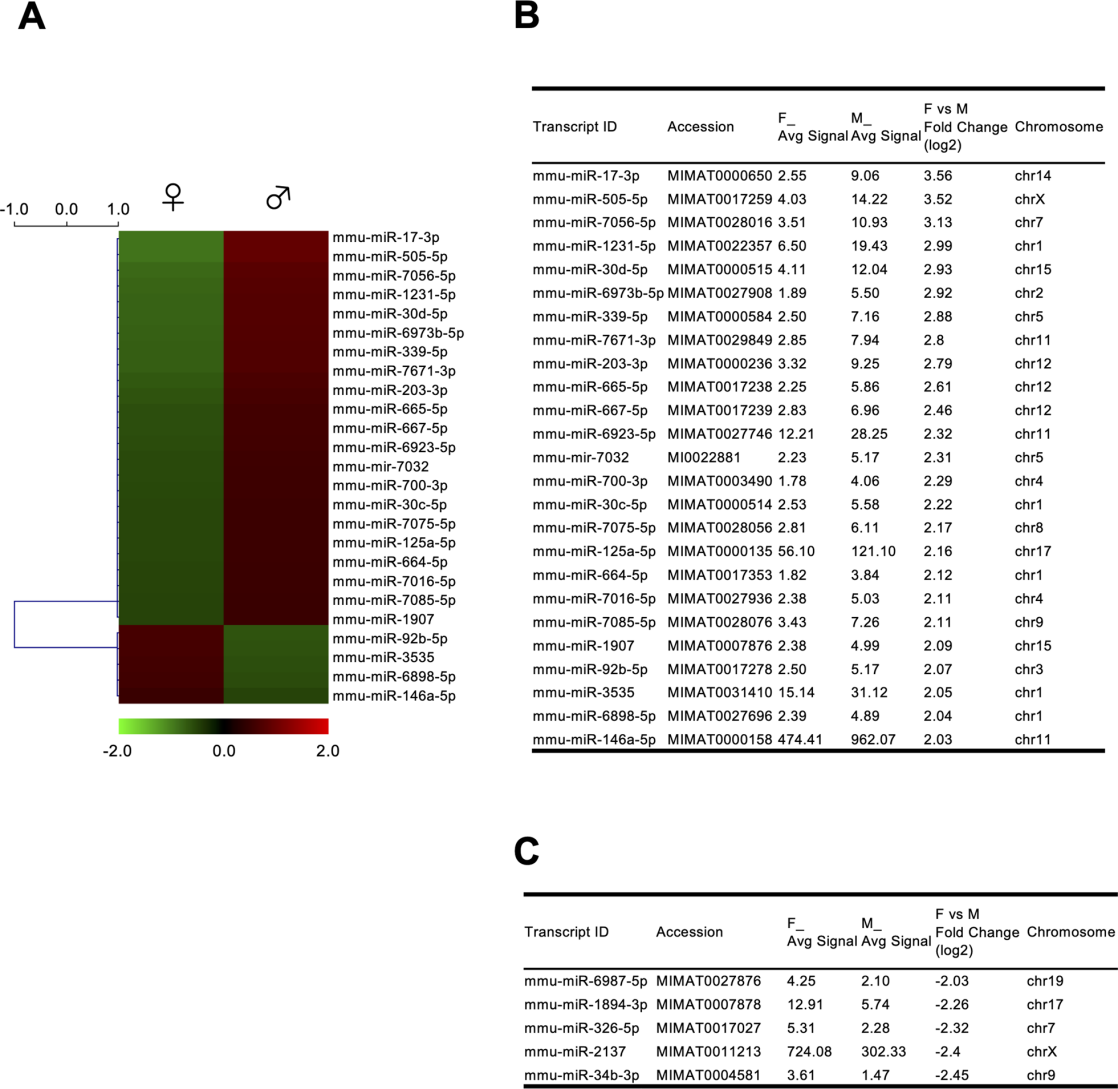
Sex-dependent miRNA expression in adult mouse hippocampal microglia. **A** Heatmap depicting microRNA (miRNA) expression profiles from microarray analysis of microglia isolated from the hippocampal tissue pooled from 8-month-old male and female mice. The heatmap highlights 30 miRNAs with differential expression between females and males, with a fold change greater than 2. **B** and** C** Summary of male-enriched (**B**) and female-enriched (**C**) miRNAs as in (**A**). Average signal intensities for females (F_Avg Signal) and males (M_Avg Signal), fold change between sexes, and the chromosomal location of each miRNA are shown

To determine the differences in miRNA expression profiles between male and female microglia, microglia isolated from the hippocampus of adult mice were subjected to miRNA microarray analysis. The resulting heatmap shows distinct differences in miRNA expression levels between males and females (Fig. 1A). In total, 30 miRNAs that were differentially expressed (|logFC|> 2) between the sexes were identified, with 25 miRNAs upregulated in males and five in females (Fig. 1B and C). Only two of the differentially expressed miRNAs were encoded by the X chromosome: the male-enriched miRNA mmu-miR-505-5p and the female-enriched miRNA mmu-miR-2137 (Fig. 1B and C).

**Fig. 2:**
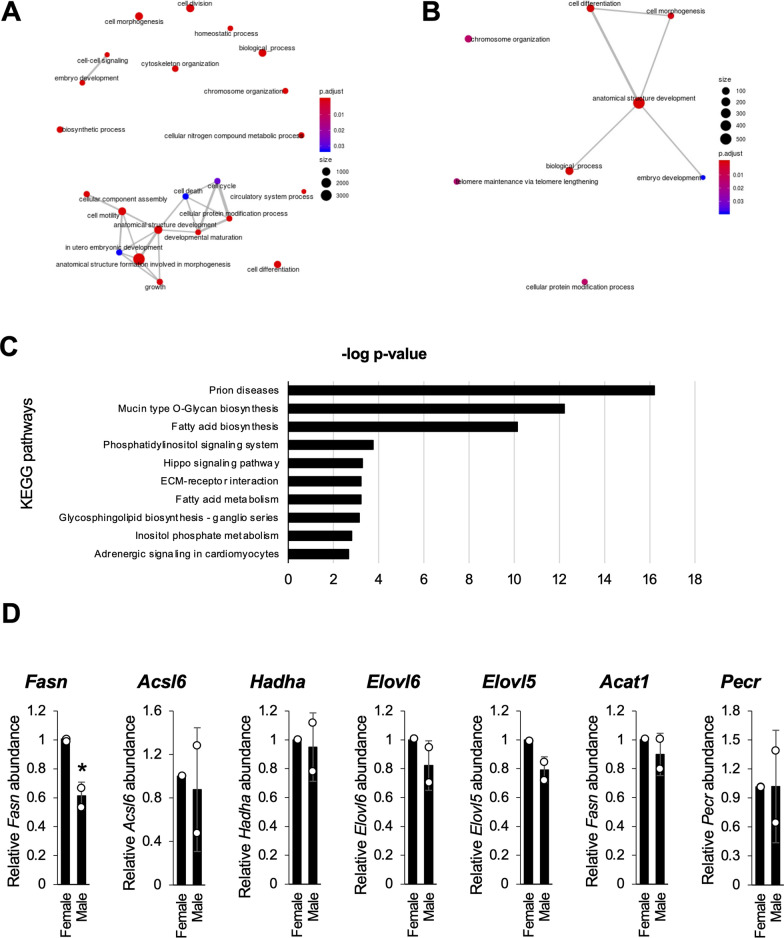
Male-enriched miRNAs regulated fatty acid synthesis-related genes. **A** and **B.** EMA-Plot representing the significant biological processes identified by Gene Ontology term analysis in male-enriched miRNAs (**A**) and female-enriched miRNAs (**B**). The genes and corresponding miRNAs targeting these processes are listed in Table S3. **C** Kyoto Encyclopedia of Genes and Genomes pathway enrichment analysis of differentially expressed miRNAs enriched in male versus female microglia. The log p-values of the top 10 pathways are shown. The full pathway list is provided in Supplementary Table S5. Significance was calculated using MirPath’s Fisher meta-analysis method. **D** Quantitative PCR (qPCR) analyses of genes related to fatty acid synthesis and metabolism. Each column represents the mean ± SD of n = 2 samples per sex, each containing pooled hippocampal microglia from five mice. *p < 0.05 versus the corresponding value for females according to Student’s *t*-test

To elucidate the functional implications of these miRNAs, a GO analysis was conducted on their predicted target genes. For the male-enriched miRNAs, 22 GO categories were enriched at a significance threshold of p < 0.05 (Fig. 2A, Supplementary Table S3), with only eight GO terms identified for the female-enriched miRNAs (Fig. 2B, Supplementary Table S3). The KEGG pathway analysis revealed enrichment in eight pathways for the target genes of female-enriched miRNAs (Supplementary Table S3) and 42 pathways for that of the male-enriched miRNAs (Supplementary Table S5, with the top 10 pathways shown in Fig. 2C), highlighting distinct sex differences in the biological processes potentially regulated by the differentially expressed miRNAs. These findings suggest that sex-dependent differences exist in miRNA-mediated biological processes, with males exhibiting greater changes. In subsequent experiments, we focused on the male-enriched miRNAs to explore their potential regulatory roles.

Among the KEGG pathways significantly enriched in the target genes of male-enriched miRNAs, the fatty acid biosynthesis pathway was the primary focus. This pathway indicated a downregulation of the expression of genes associated with fatty acid synthesis in male microglia (Fig. 2C). To investigate this further, qPCR analysis was conducted to examine the expression levels of all the fatty acid-related genes predicted to be targets of miRNAs enriched in male microglia (Supplementary Table S6). Notably, the expression of *Fasn* was observed to be significantly lower in microglia isolated from the adult male hippocampus (Fig. 2D).

### Testosterone upregulated the expression levels of miRNAs targeting fatty acid synthesis-related genes

**Fig. 3:**
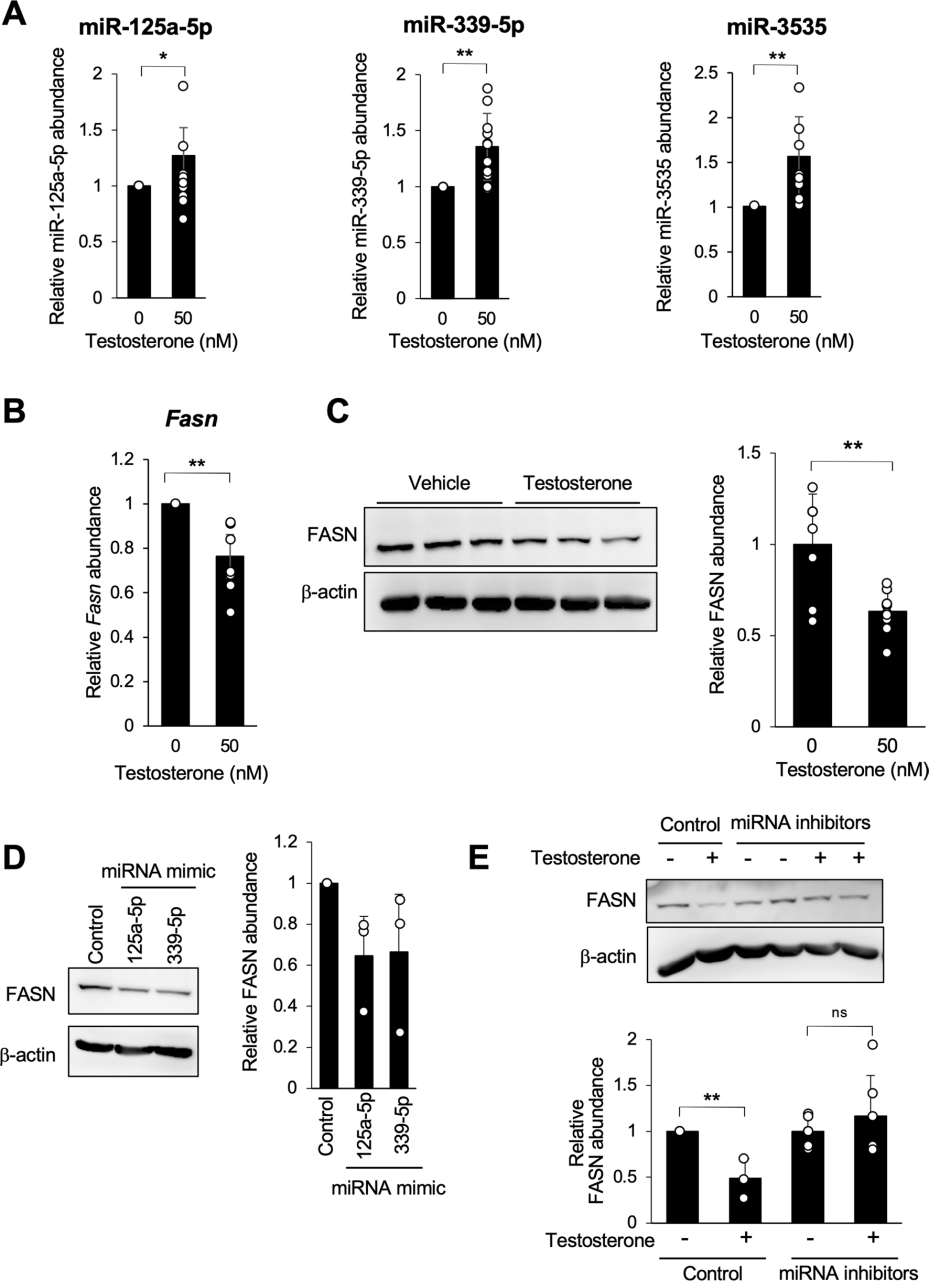
Testosterone stimulation upregulated the expression of certain male-enriched miRNAs in MG6 cells. **A** Expression of male-enriched miRNAs in MG6 cells was quantified using qPCR after 24 h of testosterone stimulation. Data represent means ± SD from 8 to 10 independent experiments. **B** MG6 cells were stimulated with testosterone for 24 h, and *Fasn* expression was quantified using qPCR. **C** FASN expression in MG6 cells was quantified using western blot after 48 h of testosterone stimulation. Representative blots from three independent experiments and the relative intensity of FASN normalized to β-actin are shown. **D** MG6 cells were transfected with control or indicated miRNA mimics and FASN expression was quantified using western blot after 48 h. Representative blots from three independent experiments and the relative intensity of FASN normalized to β-actin are shown. **E** MG6 cells were transfected with control or inhibitors of miR-125a-5p and miR-339-5p. At 24 h post-transfection, the cells were stimulated with testosterone at the final concentration of 50 nM, and FASN expression was quantified using western blot after 48 h. Representative blots from three independent experiments and the relative intensity of FASN normalized to β-actin are shown. Data represent mean ± SD from three independent experiments. *p < 0.05, **p < 0.01 versus the corresponding value for the control according to the Mann‒Whitney *U* test for two-group comparisons or one-way analysis of variance followed by Tukey–Kramer’s HSD test for multiple comparisons. Full membrane images are depicted in Supplementary Fig. S1

Given that sex hormones are the major determinants of sex-specific phenotypes [1, 8], we investigated the potential regulatory role of testosterone on the expression of male-enriched miRNAs. The effect of testosterone on the expression of male-enriched miRNA, particularly those targeting genes related to fatty acid synthesis (Supplementary Table S6), was assessed using MG6 mouse microglial cells. After 24 h of testosterone stimulation, the expression of miR-125a-5p, miR-339-5p, and miR-3535 was notably induced (Fig. 3A). Additionally, testosterone stimulation significantly reduced both the *Fasn* gene expression (Fig. 3B) and the FASN protein level (Fig. 3C). Transfection of miRNA mimics of miR-125a-5p and miR-339-5p also markedly decreased FASN expression, suggesting the involvement of testosterone-induced miRNAs in the reduced expression of FASN in male microglia (Fig. 3D). Conversely, a mimic of miR-3535 did not affect FASN protein expression levels (Supplementary Fig. S1B). When MG6 cells were transfected with inhibitors of miR-125a-5p and miR-339-5p, testosterone treatment no longer suppressed FASN expression, confirming that upregulation of these miRNAs by testosterone leads to FASN downregulation (Fig. 3E).

### Testosterone-induced suppression of FASN expression mitigated the NF-κB inflammatory signaling pathway

**Fig. 4:**
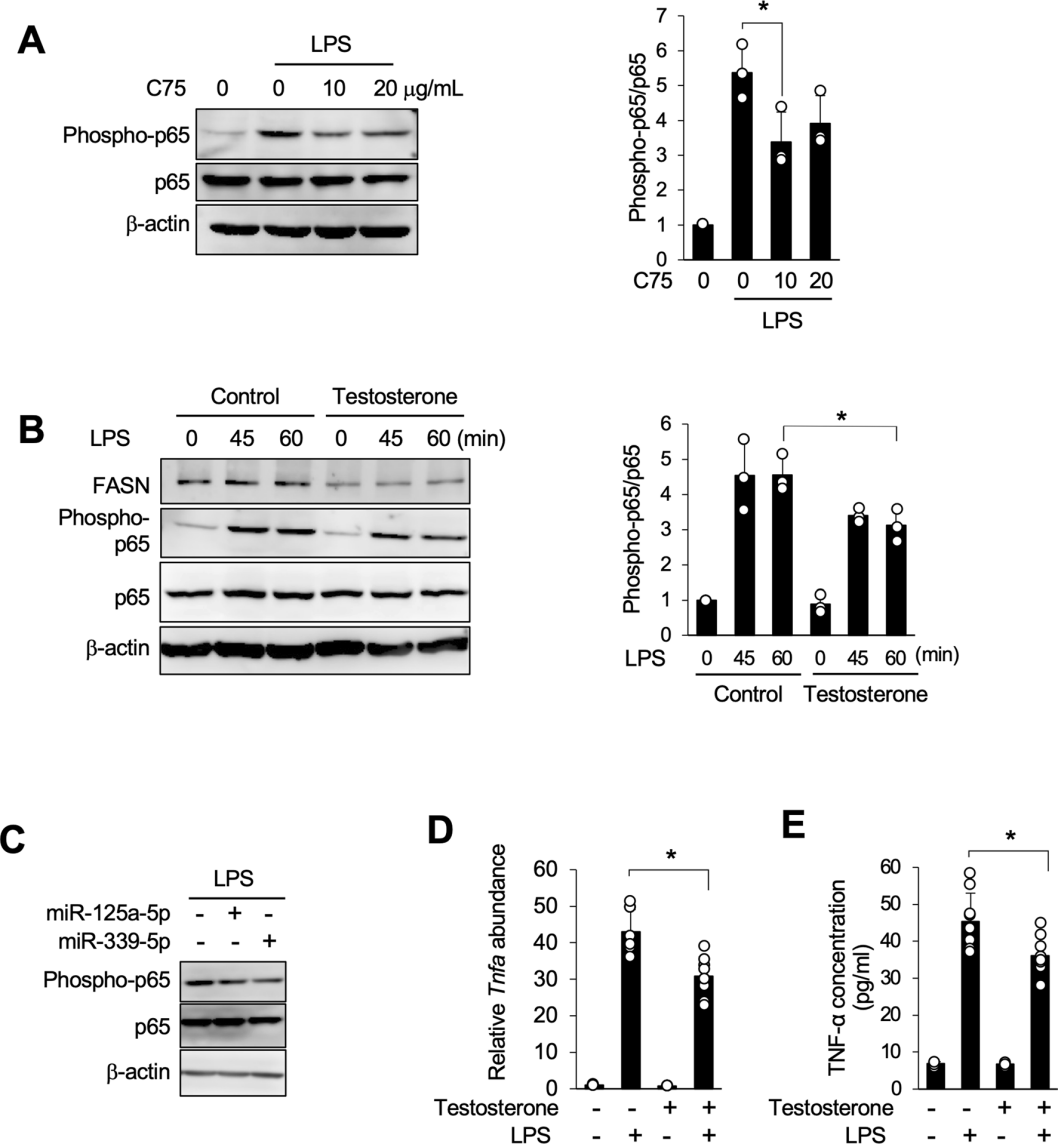
Testosterone-mediated FASN suppression inhibited NF-κB activation. **A** MG6 cells were stimulated with lipopolysaccharide (LPS) for 45 min in the presence or absence of C75 at the indicated concentrations. p65 phosphorylation was analyzed using western blot. The graph on the right shows the phosphorylated/total protein ratio of p65. **B** MG6 cells were stimulated with LPS for 45 min in the presence or absence of 50 nM testosterone. p65 phosphorylation was analyzed using western blot. Representative blots and the phosphorylated/total protein ratio of p65 are shown. **C** MG6 cells were transfected with control miRNA and indicated miRNA mimics. After 48 h, cells were stimulated with LPS for 45 min, and p65 phosphorylation was analyzed using western blot. Representative blots and the phosphorylated/total protein ratio of p65 are shown. **D** MG6 cells were stimulated with LPS for 2 h in the presence or absence of 50 nM testosterone. *Tnfa* expression was analyzed using qPCR. Data are expressed as mean ± SD from three independent experiments. **E** Secretion of TNF-α after 24 h of LPS stimulation in the presence or absence of testosterone was measured using enzyme-linked immunosorbent assay. Data are expressed as mean ± SD from three independent experiments. Full membrane images are depicted in Supplementary Figures S2–S4. *p < 0.05 versus the corresponding value for the indicated comparison according to one-way analysis of variance followed by Tukey–Kramer’s HSD test

The activation of Toll-like receptor 4 (TLR4) in microglia plays a critical role in neuroinflammation [22]. Therefore, the effect of the suppression of FASN expression on inflammatory signaling pathways in MG6 cells was examined further by assessing the potential of C75 to mitigate LPS-induced inflammation. LPS stimulation resulted in the phosphorylation of p65; however, inhibiting FASN activity using C75 significantly attenuated this response (Fig. 4A). Similarly, testosterone-induced inhibition of FASN expression also attenuated p65 phosphorylation following LPS stimulation in MG6 cells (Fig. 4B). Moreover, the inhibition of FASN expression by miRNA mimics of miR-125a-5p and miR-339-5p also suppressed LPS-induced p65 phosphorylation (Fig. 4C). Additionally, testosterone significantly suppressed the expression of *Tnfa* and the subsequent secretion of TNF-α into the culture medium after LPS stimulation (Fig. 4D and E). These findings suggest that testosterone-induced miRNAs may provide neuroinflammatory protection by modulating the NF-κB signaling pathway through suppression of FASN activity.

## Discussion

Our study revealed significant sex-dependent differences in miRNA expression profiles in the hippocampal microglia of adult mice. In this study, adult wild-type mice rather than AD model mice were used to investigate the differences in the basal state between male and female mice, aiming to identify factors that might contribute to the lower susceptibility to AD observed in males. In total, 30 miRNAs that were differentially expressed between males and females were identified, with the majority upregulated in males. This finding aligns with previous research [16] suggesting that miRNAs play crucial roles in regulating gene expression in a sex-specific manner, which may explain the sex disparities observed in AD.

The cerebral cortex and hippocampus are pivotal brain regions closely associated with cognitive function in neurodegenerative diseases [23]. Variations in miRNA characteristics are evident across different brain regions, with studies demonstrating that hippocampal microglia exhibit greater immunological activity than those in other brain regions [24]. Given that hippocampal atrophy is a key marker of AD, investigating the hippocampus is particularly important [25, 26]. Owing to the limited number of microglia that can be isolated from the hippocampus, tissue was pooled from five mice to create a single sample, resulting in the analysis of only one pooled sample. Despite this limitation, our findings suggest a potential role for miRNAs in sex-specific microglial function.

KEGG pathway analyses revealed that the target genes of male-enriched miRNAs are predominantly associated with fatty acid synthesis and metabolism pathways. This finding is particularly noteworthy, as metabolic processes, including lipid metabolism, have been implicated in the pathogenesis of neurodegenerative diseases such as AD [27]. qPCR analyses of isolated microglia revealed a specific downregulation of FASN mRNA expression in male mouse microglia. Sex steroid hormones are key determinants of sex differences in diseases [1, 8]. Notably, our study demonstrated that testosterone stimulation in MG6 cells induced the expression of several miRNAs enriched in male microglia while significantly reducing FASN expression at both the gene and protein levels. These findings suggest that testosterone plays a pivotal role in these regulatory mechanisms. Although miR-125a-5p and miR-339-5p modulated FASN expression, miR-3535 did not affect FASN expression despite being upregulated by testosterone and predicted to target *Fasn*. The target genes of miRNAs were computationally predicted based on information such as 3′UTR target sequences, number of target sites within the same 3′UTR, and target site accessibility, employing a machine learning strategy [28]. However, even if computational predictions suggest binding, the actual binding affinity or target site accessibility may differ. Consequently, the miRNA mimic might not be able to recognize the target sequence well enough to mediate gene suppression.

A decrease in androgen levels in males has been linked to increased susceptibility to AD [29]. Although the classical nuclear androgen receptor is not detected in adult microglia [30], the expression of the nongenomic testosterone receptor G protein-coupled receptor class C group 6 subtype A (GPRC6A) was confirmed [31]. This finding aligns with the findings of a study involving mice with nonfunctional androgen receptors, which demonstrated that testosterone suppresses FASN expression in hepatocytes independent of the classical nuclear androgen receptor [32]. Similarly, several studies have demonstrated that estradiol exhibits neuroprotective effects, with a decrease in estrogen levels owing to menopause associated with an increased risk of developing AD in females [33]. Microglia express the estrogen receptors ERα and ERβ, which counteract LPS-induced activation of inflammatory responses [34]. Estrogens modulate the NF-κB inflammatory signaling pathway, leading to a reduction in the production of inflammatory cytokines [35, 36]. Notably, aromatase, the enzyme that converts testosterone into estradiol, is expressed in microglia and other cell types in the brain, including neurons [37]. This raises the possibility that aromatase may convert testosterone to estradiol locally. Given that the effect of estrogens on microglial miRNAs was not assessed in this study, further research is necessary to explore the potential effects of estrogens on microglial miRNA regulation and their role in inflammation.

Suppression of FASN expression has been demonstrated to induce a protective effect against AD-related toxicities in mouse models [38]. The inhibition of FASN expression has also been demonstrated to play a protective role against inflammation [39, 40]. Palmitoylation of the myeloid differentiation primary response protein (MYD88), a key adaptor protein in the TLR family, is critical for recruiting interleukin-1 receptor-associated kinase 4 (IRAK4) and initiating downstream NF-κB signaling pathway [39]. This palmitoylation process is dependent on endogenous fatty acids synthesized by FASN [39]. Given the importance of MYD88 palmitoylation for IRAK4 recruitment, decreased FASN expression may disrupt the formation of the MYD88-IRAK4 complex, thereby impairing inflammatory signaling. Additionally, a previous study demonstrated that metformin alleviates LPS-induced inflammation in macrophages by inhibiting FASN-dependent fatty acid synthesis, thereby suppressing AKT palmitoylation and downregulating inflammatory responses [41]. In our study, similar anti-inflammatory effects were observed with the inhibition of de novo lipid synthesis by C75, further underscoring the crucial role of fatty acids in inflammation regulation. Moreover, the suppression of FASN expression by testosterone-induced miRNAs also reduced LPS-induced p65 phosphorylation and the release of inflammatory cytokines, indicating a potential neuroprotective effect.

Studies have demonstrated differential expression of miRNAs in human patients with AD [42–44]; however, these studies did not differentiate between males and females. The number of studies incorporating both sexes is relatively limited, with even fewer studies using brain tissues [45]. In our previous study, alterations were observed in the expression of specific miRNAs in the blood and brain tissues of male and female patients with AD, highlighting certain miRNAs that are differentially expressed between the sexes [45]. These results suggest that miRNAs may play a role in the pathogenesis of AD, with variations in miRNA expression between the sexes potentially contributing to the differences observed in the pathogenesis of AD between males and females. Data from mouse models indicate that miRNAs in microglia are involved in tau pathology [16]. Our current findings support the theory that high testosterone levels in male mice contribute to the differential expression of microglial miRNAs. By suppressing neuroinflammation, testosterone may play a role in reducing AD susceptibility in males.

### Perspectives and significance

Our study highlights the sex-specific regulation of miRNAs and demonstrates the effect of testosterone-induced miRNAs on microglial function, which may explain the observed sex differences in neuroinflammatory responses. The suppression of FASN expression was identified as a key modulator of NF-κB signaling, highlighting the crucial role of lipid metabolism in inflammation. The neuroprotective effects caused by the suppression of microglial inflammation could play a role in preventing neuronal damage in neurodegenerative disorders. Overall, these findings enhance the understanding of sex differences and the effect of sex hormones on immune responses, providing a basis for future research on the role of sex in AD.

## Conclusions

Significant sex differences in microglial miRNA expression patterns were observed, with certain miRNAs being regulated by testosterone, leading to the suppression of FASN expression and subsequent inhibition of the NF-κB inflammatory signaling pathway. These findings suggest that testosterone modulates inflammation in male microglia by regulating fatty acid synthesis, potentially contributing to the sex differences observed in AD pathogenesis.

## Supplementary Information

Supplementary material 1.

Supplementary material 2.

## Data Availability

The datasets used and/or analyzed during the current study are available from the corresponding author upon request. The miRNA microarray datasets generated during the current study are available in the Gene Expression Omnibus database, NCBI (GSE accession: GSE278173).
