# Supplementary material for: Sex differences in the neuroinflammatory signaling pathway: effect of miRNAs on fatty acid synthesis in microglia
Source: Biol Sex Differ. 2025 Feb 4;16:9. doi: 10.1186/s13293-025-00686-8 (PMC11792555; doi:10.1186/s13293-025-00686-8)
Supplement: Supplementary file 1 — Supplementary material 1. [file 13293_2025_686_MOESM1_ESM.zip › New folder/Additional File 1 Figure Legends.docx]

**Additional File 1**

**Supplementary Figure Legends**

**Supplementary Figure S1. FASN expression levels in MG6 cells. (A)** Full membrane images of the western blot from MG6 cells stimulated with testosterone for 48 h, as shown in Figure 3C. (**B, C**) MG6 cells were transfected with control or miRNA-3535 mimic. After 48 h, FASN expression was quantified using western blotting. Representative blots from three independent experiments (B) and full membrane images (C) are shown. **(D)** Full membrane images of the western blot band image from MG6 cells transfected with control or indicated miRNA mimics, as shown in Figure 3D. (**E**) Full membrane images of the western blot band image from MG6 cells transfected with control or inhibitors of miR-125a-5p and miR-339-5p, as shown in Figure 3E. Cropped areas are represented in a dotted square, and the molecular weight sizes (kDa) for protein markers are labeled with numbers.

**Supplementary Figure S2. Western blot images of C75-treated MG6 cells.** Full membrane images of western blots from MG6 cells stimulated with LPS for 45 min in the presence or absence of C75 at the indicated concentrations, as shown in Figure 4A. Arrowheads represent each detected band. Cropped areas are represented in a dotted square, and the molecular weight sizes (kDa) for protein markers are labeled with numbers.

**Supplementary Figure S3. Western blot images of testosterone-treated MG6 cells.** Full membrane images of western blots from MG6 cells stimulated with LPS for 45 min in the presence or absence of 50 nM testosterone, as shown in Figure 4B. Arrowheads represent each detected band. Cropped areas are represented in a dotted square, and the molecular weight sizes (kDa) for protein markers are labeled with numbers.

**Supplementary Figure S4. Western blot images of MG6 cells transfected with miRNA mimics.** Full membrane images of MG6 cells transfected with control miRNA and indicated miRNA mimics, as shown in Figure 4C. After 48 h, cells were stimulated with LPS for 45 min, and p65 phosphorylation was analyzed using western blotting. Cropped areas are represented in a dotted square, and the molecular weight sizes (kDa) for protein markers are labeled with numbers.
