# Supplementary figures and images for: Sex differences in the neuroinflammatory signaling pathway: effect of miRNAs on fatty acid synthesis in microglia
Source: Biol Sex Differ. 2025 Feb 4;16:9. doi: 10.1186/s13293-025-00686-8 (PMC11792555; doi:10.1186/s13293-025-00686-8)

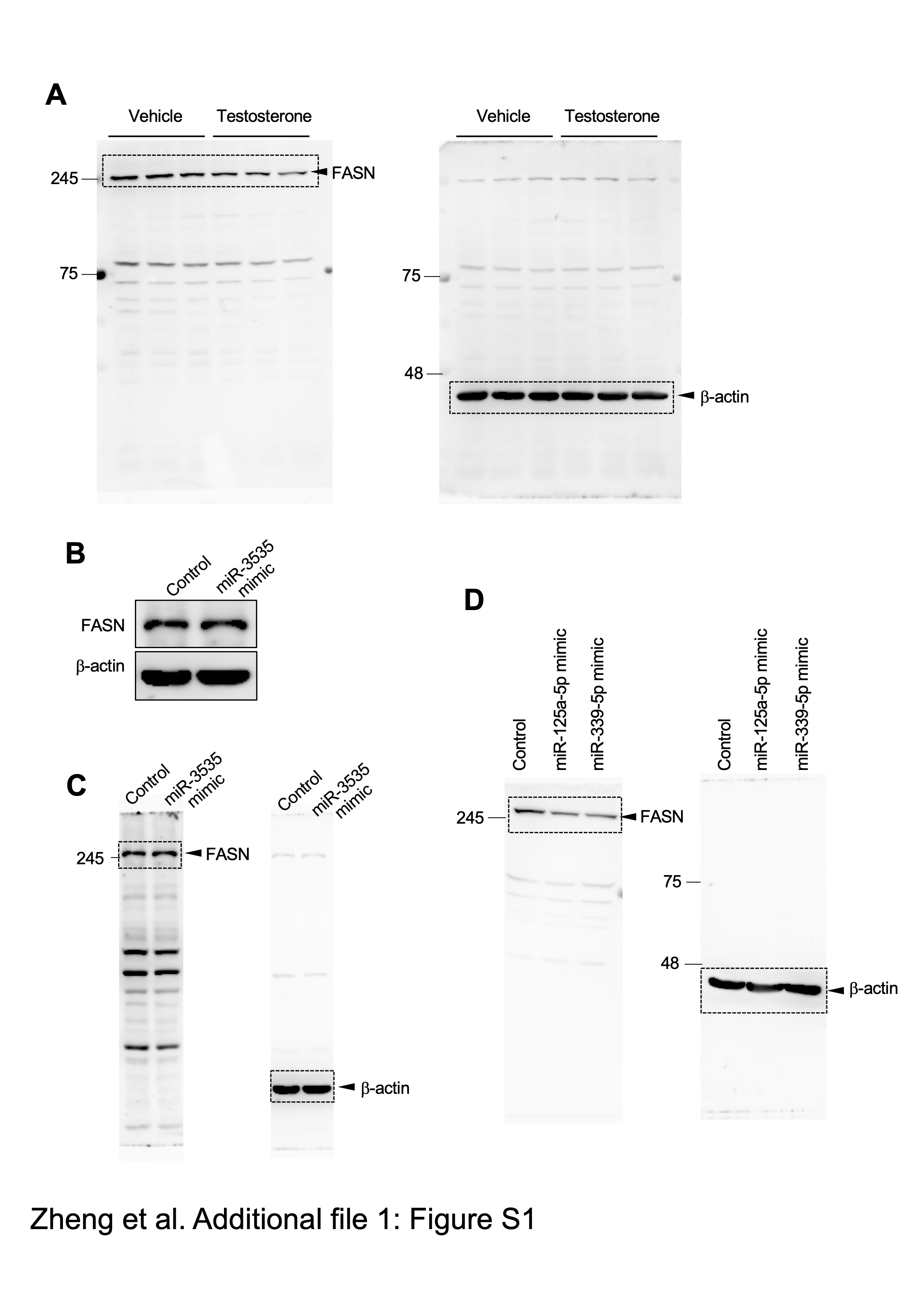

Supplement: Supplementary file 1 — Supplementary material 1. [file 13293_2025_686_MOESM1_ESM.zip › New folder/Zheng_Additional_File_1_Fig_S1.tiff]

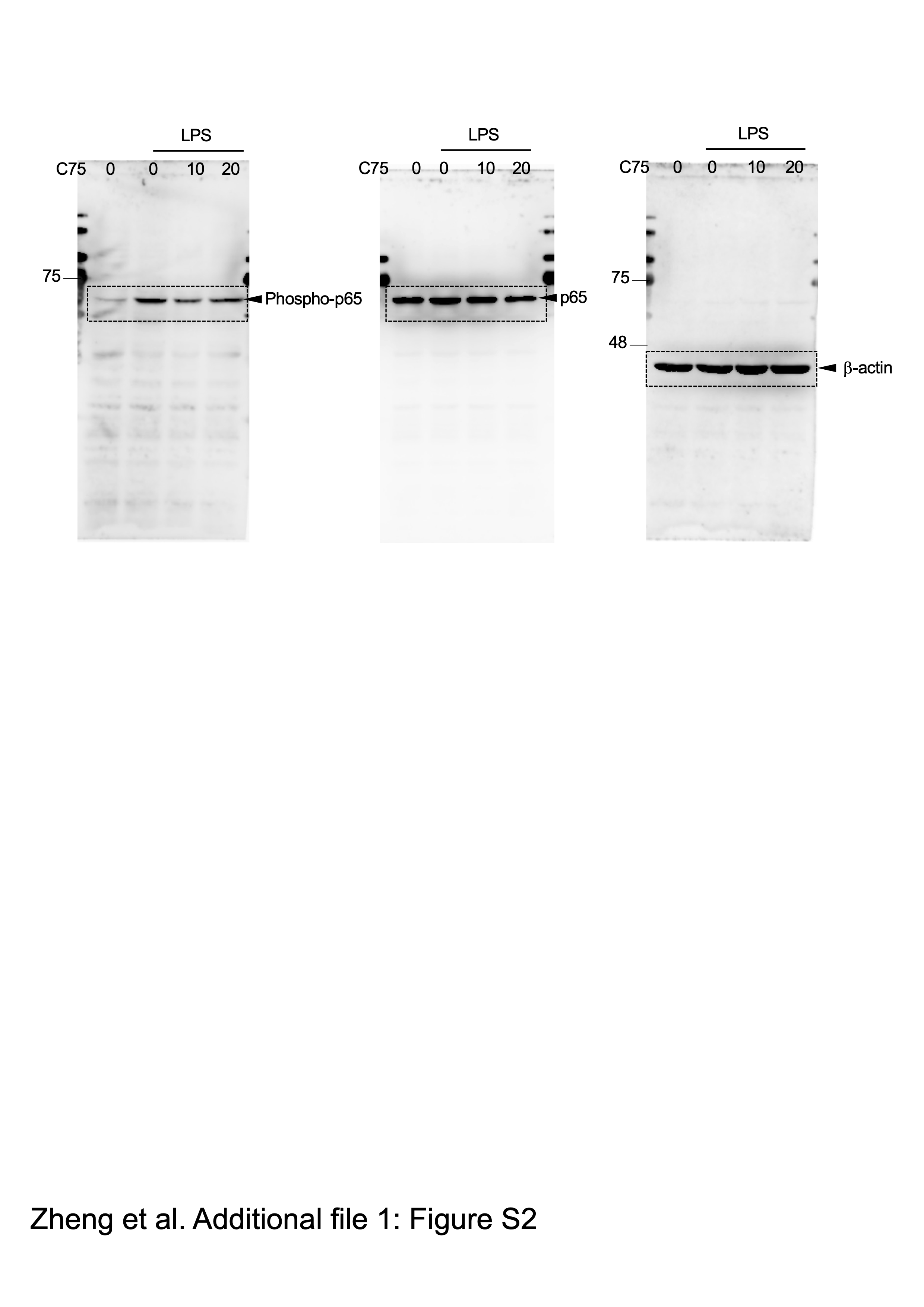

Supplement: Supplementary file 1 — Supplementary material 1. [file 13293_2025_686_MOESM1_ESM.zip › New folder/Zheng_Additional_File_1_Fig_S2.tiff]

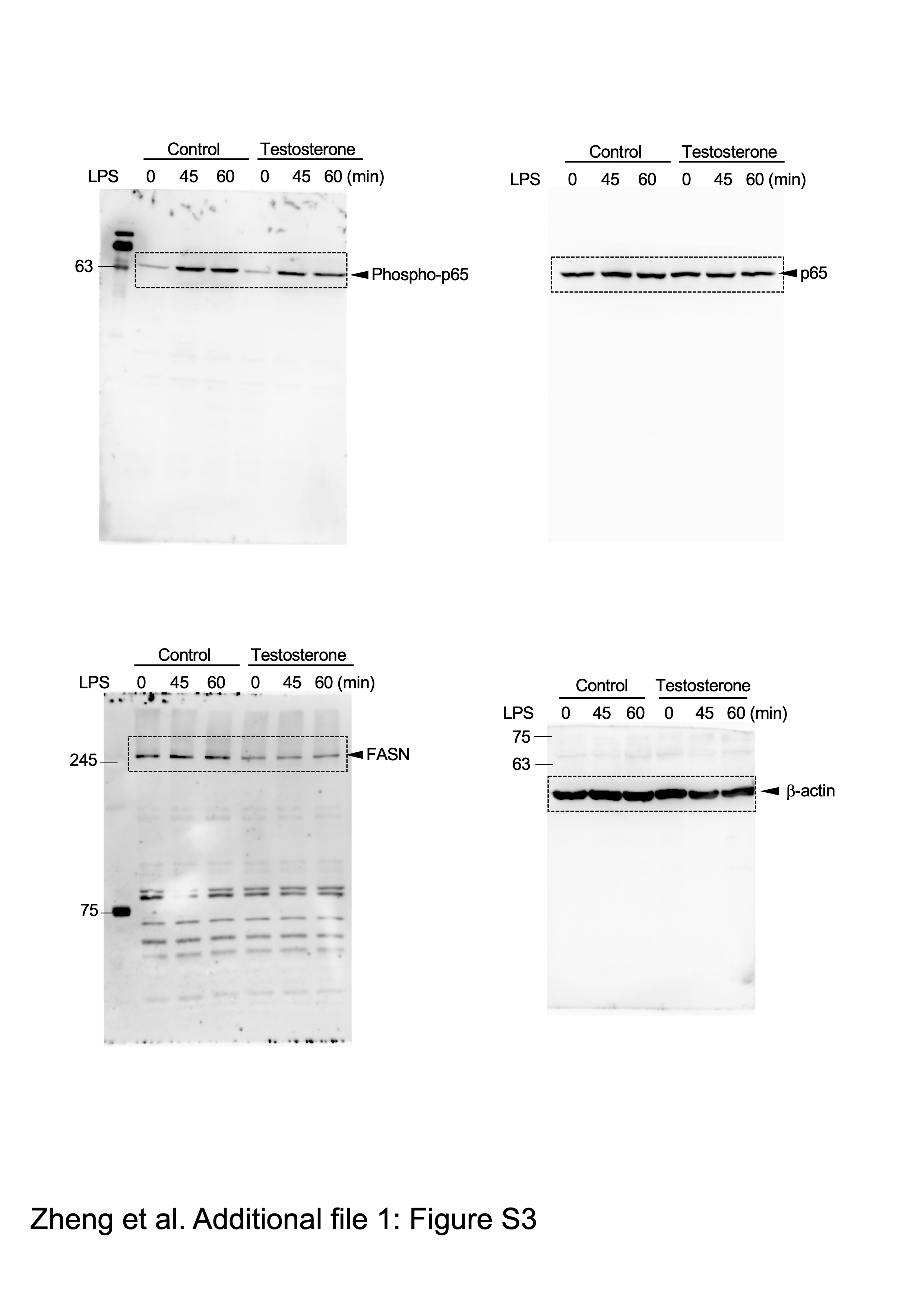

Supplement: Supplementary file 1 — Supplementary material 1. [file 13293_2025_686_MOESM1_ESM.zip › New folder/Zheng_Additional_File_1_Fig_S3.tiff]

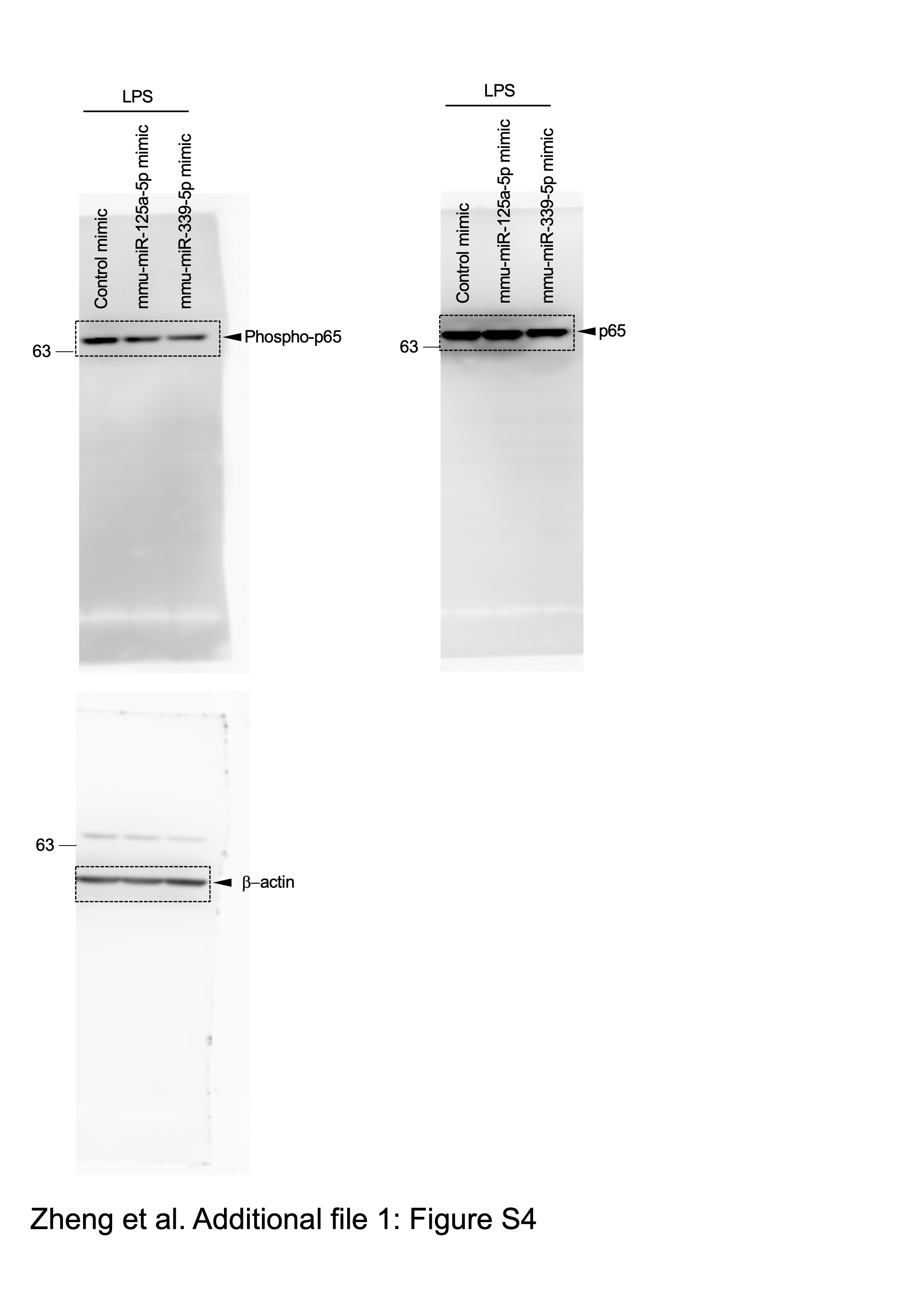

Supplement: Supplementary file 1 — Supplementary material 1. [file 13293_2025_686_MOESM1_ESM.zip › New folder/Zheng_Additional_File_1_Fig_S4.tiff]
