# Supplementary material for: Sex differences in the neuroinflammatory signaling pathway: effect of miRNAs on fatty acid synthesis in microglia
Source: Biol Sex Differ. 2025 Feb 4;16:9. doi: 10.1186/s13293-025-00686-8 (PMC11792555; doi:10.1186/s13293-025-00686-8)
Supplement: Supplementary file 2 — Supplementary material 2. [file 13293_2025_686_MOESM2_ESM.docx]

| Gene |  | Sequence (5'-3') | GenBank Accession | PrimerBank ID |
| --- | --- | --- | --- | --- |
| *Fasn* | F | GGAGGTGGTGATAGCCGGTAT | NM_007988 | 30911099a1 |
|  | R | TGGGTAATCCATAGAGCCCAG |  |  |
| *Acsl6* | F | TCCGAGACAGGACGATGTG | NM_001033599 | 28628069a1 |
|  | R | AGACTGGATCACTCTCTCAAACA |  |  |
| *Hadha* | F | GGAGGTGGTGATAGCCGGTAT | NM_178878 | 142357440c1 |
|  | R | TGGGTAATCCATAGAGCCCAG |  |  |
| *Elovl6* | F | GGAGGTGGTGATAGCCGGTAT | NM_130450 | 18496985a1 |
|  | R | TGGGTAATCCATAGAGCCCAG |  |  |
| *Elovl5* | F | GGAGGTGGTGATAGCCGGTAT | NM_134255 | 258679505c2 |
|  | R | TGGGTAATCCATAGAGCCCAG |  |  |
| *Acat1* | F | GGAGGTGGTGATAGCCGGTAT | NM_144784 | 118130035c1 |
|  | R | TGGGTAATCCATAGAGCCCAG |  |  |
| *Pecr* | F | GGTCAGAGCTACCTAGCGG | NM_023523 | 31980804a1 |
|  | R | GACCACGTTACACCCCAGG |  |  |
| *Tnfa* | F | CAGGCGGTGCCTATGTCTC | NM_013693 | 133892368c1 |
|  | R | CGATCACCCCGAAGTTCAGTAG |  |  |
| *18s rRNA* | F | GTAACCCGTTGAACCCCATT | NR_003278 |  |
|  | R | CCATCCAATCGGTAGTAGCG |  |  |

**Supplementary Table S1. Sequences of qPCR primers used for gene expression analysis**

This table lists the sequences of PCR primers (F, forward; R, reverse) for the indicated genes. GenGank Accession and PrimerBank IDs (<https://pga.mgh.harvard.edu/primerbank/index.html>) are indicated.

| Name | Cat# | Manufacturer |
| --- | --- | --- |
| FASN | 10624-2-AP | Proteintech |
| NF-κB p65 (D14E12) | 8242 | Cell Signaling Technology |
| Phospho-NF-κB p65 (Ser536) (93H1) | 3033 | Cell Signaling Technology |
| β-actin (AC-15) | A1978 | Sigma-Aldrich |
| Anti-rabbit HRP | 7074 | Cell Signaling Technology |
| Anti-mouse HRP | 7076 | Cell Signaling Technology |

**Supplementary Table S2. Antibodies used for immunoblot analysis**

|  | GO Category | *p*-value | #genes | #miRNAs |
| --- | --- | --- | --- | --- |
| Male | anatomical structure development | 7.852E-159 | 949 | 23 |
|  | biological_process | 7.085E-143 | 3773 | 23 |
|  | cell differentiation | 2.4334E-93 | 722 | 23 |
|  | embryo development | 3.9889E-48 | 268 | 23 |
|  | cell morphogenesis | 3.1787E-28 | 199 | 22 |
|  | anatomical structure formation involved in morphogenesis | 1.365E-27 | 215 | 23 |
|  | cellular protein modification process | 4.6273E-20 | 510 | 23 |
|  | cellular nitrogen compound metabolic process | 1.1274E-19 | 910 | 23 |
|  | chromosome organization | 4.7428E-18 | 148 | 21 |
|  | biosynthetic process | 3.671E-17 | 798 | 23 |
|  | developmental maturation | 7.1292E-11 | 52 | 21 |
|  | homeostatic process | 1.8299E-09 | 201 | 23 |
|  | cytoskeleton organization | 8.2057E-09 | 172 | 22 |
|  | cell motility | 7.8609E-08 | 142 | 20 |
|  | cell-cell signaling | 8.8658E-05 | 142 | 22 |
|  | circulatory system process | 0.00010323 | 44 | 20 |
|  | cellular component assembly | 0.0001331 | 252 | 23 |
|  | cell division | 0.00022355 | 110 | 21 |
|  | growth | 0.00042823 | 95 | 23 |
|  | cell cycle | 0.02808264 | 200 | 22 |
|  | in utero embryonic development | 0.03291681 | 85 | 22 |
|  | cell death | 0.03300671 | 172 | 22 |
| Female | biological_process | 1.0436E-18 | 549 | 5 |
|  | anatomical structure development | 3.1576E-13 | 122 | 5 |
|  | cell differentiation | 2.6201E-09 | 99 | 5 |
|  | cell morphogenesis | 0.00101175 | 31 | 5 |
|  | chromosome organization | 0.01351783 | 24 | 4 |
|  | cellular protein modification process | 0.01351783 | 77 | 5 |
|  | telomere maintenance via telomere lengthening | 0.01543837 | 4 | 2 |
|  | embryo development | 0.03879032 | 30 | 4 |

**Supplementary Table S3. GO categories enriched in target genes of male- and female-enriched miRNAs**

GO pathways enriched in the target genes of male- and female-enriched miRNAs are shown. The table includes the p-value, the number of genes involved in each GO category, and the number of corresponding miRNAs that target these genes.

| KEGG pathway | *p*-value | #genes | #miRNAs |
| --- | --- | --- | --- |
| Fatty acid elongation | 2.8418E-08 | 2 | 2 |
| Thyroid hormone synthesis | 0.00109498 | 1 | 1 |
| Biosynthesis of unsaturated fatty acids | 0.00109498 | 2 | 2 |
| Morphine addiction | 0.00184615 | 6 | 3 |
| Fatty acid degradation | 0.00249604 | 1 | 1 |
| Taste transduction | 0.00300726 | 3 | 3 |
| Fatty acid metabolism | 0.00532694 | 1 | 1 |
| Calcium signaling pathway | 0.01321751 | 14 | 4 |

**Supplementary Table S4. KEGG pathways enriched in the target genes of female-enriched miRNAs**

This table presents KEGG pathways enriched in the target genes of female-enriched miRNAs, including the p-value, the number of genes involved in each pathway, and the number of corresponding miRNAs that target these genes.

| KEGG pathway | *p*-value | #genes | #miRNAs |
| --- | --- | --- | --- |
| Prion diseases | 6.3412E-17 | 10 | 7 |
| Mucin type O-Glycan biosynthesis | 5.9596E-13 | 9 | 6 |
| Fatty acid biosynthesis | 7.3238E-11 | 2 | 2 |
| Phosphatidylinositol signaling system | 0.00017577 | 29 | 14 |
| Hippo signaling pathway | 0.00052364 | 35 | 16 |
| Fatty acid metabolism | 0.00060472 | 7 | 6 |
| ECM-receptor interaction | 0.00060472 | 20 | 14 |
| Glycosphingolipid biosynthesis - ganglio series | 0.00072416 | 6 | 6 |
| Inositol phosphate metabolism | 0.00155231 | 23 | 13 |
| Adrenergic signaling in cardiomyocytes | 0.00216196 | 42 | 18 |
| Adherens junction | 0.00284658 | 25 | 12 |
| Axon guidance | 0.00284658 | 43 | 17 |
| Oocyte meiosis | 0.00391598 | 35 | 17 |
| Circadian rhythm | 0.00547851 | 13 | 12 |
| MAPK signaling pathway | 0.00547851 | 65 | 17 |
| Rap1 signaling pathway | 0.00710154 | 53 | 16 |
| Ubiquitin mediated proteolysis | 0.00710154 | 42 | 19 |
| Gap junction | 0.00891042 | 21 | 14 |
| Focal adhesion | 0.00955388 | 55 | 18 |
| cGMP-PKG signaling pathway | 0.01058563 | 48 | 18 |
| PI3K-Akt signaling pathway | 0.0175454 | 83 | 20 |
| FoxO signaling pathway | 0.01800836 | 38 | 13 |
| Long-term potentiation | 0.01800836 | 22 | 14 |
| Glioma | 0.01800836 | 18 | 14 |
| Regulation of actin cytoskeleton | 0.01800836 | 56 | 20 |
| Chronic myeloid leukemia | 0.02132101 | 21 | 13 |
| Melanoma | 0.02156449 | 21 | 13 |
| Signaling pathways regulating pluripotency of stem cells | 0.02177011 | 37 | 13 |
| Arrhythmogenic right ventricular cardiomyopathy (ARVC) | 0.02214714 | 23 | 16 |
| Calcium signaling pathway | 0.02962746 | 45 | 15 |
| Hypertrophic cardiomyopathy (HCM) | 0.02962746 | 25 | 16 |
| Proteoglycans in cancer | 0.03172993 | 46 | 17 |
| Fc gamma R-mediated phagocytosis | 0.03220807 | 26 | 14 |
| mRNA surveillance pathway | 0.03220853 | 29 | 16 |
| Thyroid hormone synthesis | 0.03244869 | 13 | 9 |
| Neurotrophin signaling pathway | 0.03498762 | 33 | 14 |
| T cell receptor signaling pathway | 0.03498762 | 29 | 16 |
| Endometrial cancer | 0.03643593 | 16 | 11 |
| Renal cell carcinoma | 0.0376922 | 19 | 13 |
| Endocytosis | 0.03928242 | 54 | 20 |
| Prostate cancer | 0.04189767 | 24 | 14 |
| Dilated cardiomyopathy | 0.0476001 | 25 | 16 |

**Table S5. Enriched target genes in the KEGG pathways of male-enriched miRNAs**

This table presents KEGG pathways enriched in the target genes of male-enriched miRNAs, including the p-value, the number of genes involved in each pathway, and the number of corresponding miRNAs that target these genes.

| KEGG pathway | *p*-value | Genes | miRNAs |
| --- | --- | --- | --- |
| Fatty acid biosynthesis | 7.3238E-11 | Fasn, Acsl6 | mmu-miR-125a-5p, mmu-miR-1907 |
| Fatty acid metabolism | 0.00060472 | Fasn, Hadha, Elovl6, Elovl5, Acat1, Pecr, Acsl6 | mmu-miR-30d-5p, mmu-miR-30c-5p, mmu-miR-125a-5p, mmu-miR-3535,  mmu-miR-1907, mmu-miR-339-5p |

**Table S6. miRNAs enriched in male microglia with potential target genes related to fatty acid synthesis and metabolism**

This table lists male-enriched miRNAs that target genes involved in fatty acid synthesis and metabolism, including the p-values, the names of genes involved in each pathway (details of two selected KEGG pathways of interest are shown in Table S4), and the corresponding miRNAs that target these genes.
